# Endplate Bone Quality Assessment for Preoperative Planning and Patient-Specific Implementation in Lumbar Spine Surgery

**DOI:** 10.3390/jcm15072800

**Published:** 2026-04-07

**Authors:** Wesley P. Jameson, Bailey D. Lupo, Andrew M. Schwartz, Andrew Daigle, Ahmed Anwar, Smith Surendran, Huy Tran, Christian Quinones, Deepak Kumbhare, Bharat Guthikonda, Stanley Hoang

**Affiliations:** Department of Neurosurgery, Louisiana State University Health Science Center-Shreveport, Shreveport, LA 71103, USA

**Keywords:** endplate bone quality, EBQ, VBQ, lumbar spine, cage subsidence, preoperative planning

## Abstract

**Background/Objectives**: Poor bone quality is strongly associated with adverse surgical events. Although dual-energy X-ray absorptiometry (DXA) remains the gold standard for bone mineral density (BMD) assessment, logistical barriers may limit its preoperative application. The Endplate Bone Quality (EBQ) score is an MRI-derived metric quantifying subchondral bone quality at the vertebral endplate with demonstrated predictive value for cage subsidence following lumbar interbody fusion. However, EBQ has been measured exclusively at the operative level in surgical cohorts. This study aimed to assess level-specific EBQ scores across the entire lumbar spine and compare distributions across age, sex and osteoporosis subgroups. **Methods**: A single-institution retrospective review of T1-weighted lumbar MRI studies from patients evaluated for lower back pain from 2020 to 2025 was performed. EBQ was independently scored by two blinded raters at each disc space from L1–L2 to L5–S1 using 3 mm endplate ROIs normalized to a CSF ROI at L3. Interrater reliability was assessed via ICC, Pearson correlation, and RMSE. Patients were stratified by age (≤60 vs. >60 years), sex, and osteoporosis status, and subgroup comparisons were performed for overall and level-specific EBQ score. **Results**: A total of 96 patients with an average age of 61.0 ± 9.42 years were included in this study. The majority of patients included were female (87.5%), and 18.8% had been diagnosed with osteoporosis. EBQ scores demonstrated a progressive caudal increase across all subgroups from L2–L3 to L5–S1. Overall interrater reliability was acceptable (ICC = 0.76), with level-specific ICCs ranging from 0.70 to 0.83. No significant differences were observed between age or sex subgroups. Osteoporotic patients demonstrated significantly higher EBQ at L1–L2, L2–L3, and overall (all *p* < 0.05), with no significant differences at L3–L4 through L5–S1. **Conclusions**: This study provides normative, level-specific EBQ reference data throughout all levels of the lumbar spine. The increase in EBQ scores seen among caudal levels and reduced osteoporotic discriminatory power support the importance of level-specific context when interpreting EBQ thresholds. These findings may support future studies evaluating threshold development for EBQ.

## 1. Introduction

Bone quality is characterized by the combination of factors that collectively determine a bone’s capacity to withstand fracture [1]. Disorders that compromise bone quality, such as osteopenia and osteoporosis, affect over 54 million individuals in the United States and possess significant implications for spinal surgery outcomes [2]. The relationship between poor bone quality and adverse postoperative events, including pedicle screw loosening, cage subsidence (CS), and pseudarthrosis, has prompted national governing bodies to recommend preoperative bone quality screening prior to lumbar spine procedures [3]. The imaging techniques developed to quantify BMD have evolved over time, with each demonstrating unique strengths, limitations and clinical applicability.

### 1.1. Photon Absorptiometry

Cameron et al. introduced Single-Photon Absorptiometry (SPA), which consists of a low-energy photon beam projected at an appendicular skeletal site [4]. By using a scintillation detector, BMD can be calculated from the attenuation of the bone being measured compared to that of the calibration standard. Building upon this work, Dual-Photon Absorptiometry (DPA) quantifies bone density using photon attenuation with two radioisotopes, allowing for measurement at axial skeletal locations [5]. However, poor image resolution, low precision and prolonged acquisition times limit the clinical applicability of DPA [6].

### 1.2. Quantitative Computed Tomography

Quantitative computed tomography (QCT) provides three-dimensional volumetric BMD measurements capable of distinguishing between trabecular and cortical bone. The high sensitivity of QCT allows for the identification of vertebral compression fractures that are not apparent on conventional imaging [7]. In the absence of trauma or malignancy, this finding is highly suggestive of osteoporosis and may support the diagnosis of patients failing to meet additional criteria [8]. Although reduced scan times have prompted exploration into the use of QCT as an opportunistic BMD screening tool, this benefit is offset by protocol discrepancies, increased costs and higher radiation dose [9,10].

### 1.3. X-Ray Absorptiometry

In single x-ray absorptiometry (SXA), bone density is calculated using photon absorption in bone, corrected by subtracting soft tissue absorption [11]. Although SXA improves upon the precision errors observed in SPA, measurements are similarly limited to the appendicular skeleton [12].

Dual-energy x-ray absorptiometry (DXA) directs heavily filtered x-rays of two differing energy levels, differentiating bone from the surrounding tissue and facilitating the accurate measurement of axial BMD [13]. Results are provided as a T-score, which compares a patient’s BMD to that of a healthy adult reference population, as well as an age-matched Z-score [14]. With a diagnostic threshold of −2.5 or lower for postmenopausal women and men 50 years of age or older, the T-score is instrumental in defining and diagnosing osteoporosis [15].

Despite its widespread adoption, DXA has important limitations. BMD measurements between different manufacturers cannot be directly compared without cross-calibration [16]. Additionally, vascular calcification and degenerative bone changes can artificially elevate BMD measurements, potentially masking true bone quality deficits [17]. Although many spine surgeons advocate for preoperative bone mineral density (BMD) imaging, patients with reduced BMD are often asymptomatic or fail to meet recommendations for targeted risk assessments [18,19,20]. These limitations have influenced researchers to explore alternatives that can be obtained from opportunistic imaging, such as those obtained during routine preoperative workflows.

### 1.4. VBQ Calculation

The Vertebral Bone Quality (VBQ) score was introduced by Ehresman et al. as an MRI-derived bone quality assessment metric [20]. The basis of VBQ is derived from well-established pathophysiologic processes associated with osteoporotic bone changes. Stress, aging and genetic predispositions may influence mesenchymal stem cell (MSC) differentiation toward adipocytes and away from osteoblasts, resulting in increased bone marrow adiposity [21]. Increased vertebral adiposity appears hyperintense on T1-weighted MRI sequences, which are routinely obtained during preoperative workflows, providing an opportunistic bone quality screening tool without additional radiation exposure.

VBQ score quantifies these changes by normalizing the vertebral signal intensity (SI) of circular regions of interest (ROI) within the trabecular bone of the L1–L4 vertebral bodies to that of the CSF posterior to L3, ensuring to avoid contact with cortical bone or the cauda equina. This normalization suggests that VBQ score may be independent of the scanner used, providing a generalizable and clinically applicable metric. Higher scores reflect greater fatty infiltration and poorer bone quality, and have demonstrated significant correlation with DXA T-scores and QCT BMD, as well as predictive value for cage subsidence (CS) [22,23]. The equation for VBQ is represented as:

$$VBQ=\frac{\left({SI}_{L1-L4}\right)}{{SI}_{CSF}}$$

### 1.5. EBQ Calculation

Although the VBQ serves as a clinically meaningful technique to assess bone quality, ROI sampling does not include regions of the vertebrae in direct contact with interbody cages [24,25]. The vertebral endplate serves as the primary load-bearing surface following vertebral interbody fusion, with loss of the integrity within this region being commonly implicated in cage subsidence. Jones et al. introduced Endplate Bone Quality (EBQ) as a site-specific metric designed to evaluate the subchondral bone of the vertebral endplate and provide information relevant to the biomechanical challenges associated with interbody fusion [26]. Similar to VBQ, higher EBQ scores indicate decreased quality of the vertebral endplate and therefore greater risk of CS due to its role in axial load bearing.

Regions of interest (ROI) were described as the subchondral bone within 3 mm of the upper end plate (UEP) and lower end plate (LEP) at the level being evaluated for operation. In addition, a rectangular-shaped ROI was placed posterior to L3 to obtain the signal intensity (SI) of the cerebrospinal fluid (CSF). Similar to VBQ, caution was taken to avoid the cauda equina and other surrounding structures. The average signal intensity of the end plates was then divided by that obtained from the CSF. This equation is represented as:

$$EBQ=\frac{({SI}_{UEP}+{SI}_{LEP})/2}{{SI}_{CSF}}$$

### 1.6. Methodological Variation in EBQ Calculation

Nine published studies have evaluated EBQ scores in the context of lumbar interbody fusion. Endplate ROI thickness was 3 mm in eight studies, while Bian et al. used a narrower 1.25 mm ROI [27]. Although the L3 level was consistently reported for CSF sampling, two studies used rectangular ROI [26,28], while seven studies substituted circular ROIs [27,29,30,31,32,33,34]. Notably, no study reported the dimensions of the CSF ROI. When the primary measurement site at L3 was obstructed, L2 or L4 was commonly used. However, one study describes sampling from a parasagittal slice, while three studies did not disclose their alternative protocol.

This methodological heterogeneity has meaningful clinical implications. Variation in ROI dimensions, shape, and CSF sampling site can potentially alter EBQ values, limiting direct threshold comparison across studies and surgical approaches. Standardizing segmentation protocols supports reproducibility and valid cross-study comparison prior to clinical implementation.

Despite the growing evidence base for EBQ, all published studies have measured the score exclusively at the operative level in surgical cohorts, and none have characterized level-specific EBQ distributions across the full lumbar spine. Without normative reference data, the meaningful interpretation of operative-level EBQ score remains challenging, limiting its utility as a patient-specific planning tool. The primary outcome of this study is to address this gap by characterizing level-specific EBQ scores from L1–L2 to L5–S1, while secondary outcomes included EBQ score comparisons for each level across age, sex, and osteoporotic subgroups.

## 2. Methods

### 2.1. Study Design and Participants

This study was approved by the Institutional Review Board of Louisiana State University Health Sciences Center–Shreveport. Patient consent was waived due to the retrospective and de-identified nature of this study. This institution’s neurosurgery imaging database was retrospectively queried for T1-weighted MRI studies of patients evaluated for lower back pain between 2020 and 2025.

The inclusion criteria for T1-weighted MRIs were an age ≥ 18 years and available comprehensive DICOM metadata. The exclusion criteria were prior spinal instrumentation or anatomy in which ROI placement was disrupted. Patient electronic health records were reviewed for medical and surgical history. All MRI scans were performed using a Siemens scanner, with field strengths of either 1.5 Tesla (55%) or 3 Tesla (45%). All included MRI studies were reviewed and confirmed by a board-certified neurosurgeon.

Patients were stratified and compared across age, sex and osteoporosis. Age groups were defined as those ≤ 60 years and those >60 years at the time of MRI acquisition. Osteoporosis was defined as a T-score ≤ −2.5 based on the lowest DXA T-score extracted from radiology reports of the lumbar spine, bilateral femoral neck and total hip.

### 2.2. Imaging Segmentation and EBQ Calculation

Manual EBQ scoring was performed on the midsagittal slice of T1-weighted lumbar MRIs. For each level, vertebral end plate ROIs encompassed the superior and inferior 3 mm of each vertebra from L1–S1, with a circular ROI corresponding to the CSF placed posterior to L3. SI was measured and collected for adjacent vertebral endplates and CSF. A parasagittal slice was used in instances where CSF measurements could not be measured on the mid-sagittal slice.

**Figure 1:**
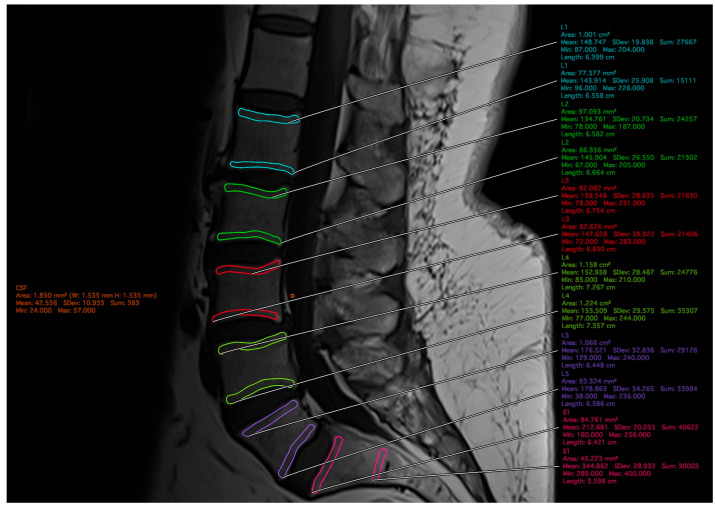
Placement of regions of interest (ROI) for each vertebra from L1–S1 and cerebrospinal fluid (CSF). The superior and inferior 3 mm of each vertebral body was used for endplate ROI placement and labeled respective to level. CSF ROI was placed posterior to L3.

In-person instruction was provided to two raters, and the quality of measurements was reviewed and confirmed by a board-certified neurosurgeon. Raters were blinded and independently measured each MRI. The SIs of the ROIs were entered on a standardized spreadsheet, and corresponding ROI placement screenshots were recorded. After data collection, all screenshots and excluded MRI studies were reviewed and confirmed by a board-certified neurosurgeon. Level-specific EBQ was calculated using the average SI between the lower endplate (LEP) of the rostral vertebra and upper end plate (UEP) of the caudal vertebra, standardized to the SI of the CSF. The average SI from LEP of L1 to UEP of S1 was also standardized to the SI of CSF to obtain the overall EBQ score. An example of ROI placement is depicted in Figure 1.

### 2.3. Statistical Analysis

The normality of the data distribution was assessed with Shapiro-Wilk tests. Descriptive statistics were calculated for demographic variables, level-specific EBQ and overall EBQ. Continuous variables are presented as means ± SD and categorical variables as counts and percentages. Interrater reliability was assessed using intraclass correlation coefficients (ICC 2,1), Pearson correlation (r), root mean square error (RMSE), and mean differences with 95% confidence intervals (CI). Independent t-tests, or Mann–Whitney U tests for non-normally distributed data, were used to assess systematic differences between measurements. RStudio (2025.09.2+418) was utilized for statistical testing, which were all two-tailed and defined as significant if *p* ≤ 0.05.

## 3. Results

### 3.1. Patients

Of the 96 patients included in this study, 84 were female (87.5%) and 12 were male (12.5%). Patient age ranged from 40–85 years, with a mean of 61.0 ± 9.42 years. Age subgroups included 43 patients less than or equal to 60 years of age and 53 patients greater than 60 years of age. Of the 18 patients with osteoporosis, 5 were younger than 60, while 13 were 60 years of age or older. A history of smoking was seen in 36 patients (37.5%). Previous medical history among the cohort included hypertension (70.8%), diabetes (30.2%), dyslipidemia (31.3%), obesity (43.2%), vitamin D deficiency (18.8%), obesity (43.2%), chronic kidney disease (12.9%), depression (16.7%), and anxiety (28.1%). (Table 1).

### 3.2. Summary of Findings

The mean EBQ scores across all levels are summarized in Table 2. No statistically significant differences were observed between age or sex (all *p* > 0.05). Osteoporotic patients demonstrated significantly higher EBQ scores overall and at the L1–L2 and L2–L3 levels (all *p* < 0.05).

### 3.3. Inter-Rater Reliability

Agreement between raters was acceptable across all lumbar spine measurements. Aggregate EBQ showed a mean difference (MD) of 0.09 (95% CI, 0.004–0.18), with a standard deviation of difference (SDD) of 0.42, RMSE of 0.30, r of 0.77 (*p* < 0.001), and an ICC of 0.76 (95% CI, 0.66–0.83). The reliability of level-specific EBQ demonstrated the lowest correlation and agreement between raters at the L3–L4 disc space (ICC = 0.70), with scores improving in the more rostral and caudal levels. The L1–L2 disc space showed the greatest agreement between raters, with an ICC of 0.83. Agreeability metrics between reviewers for aggregate and level-specific EBQ scores are shown in Table 3.

### 3.4. Age Associated EBQ Scores

**Figure 2:**
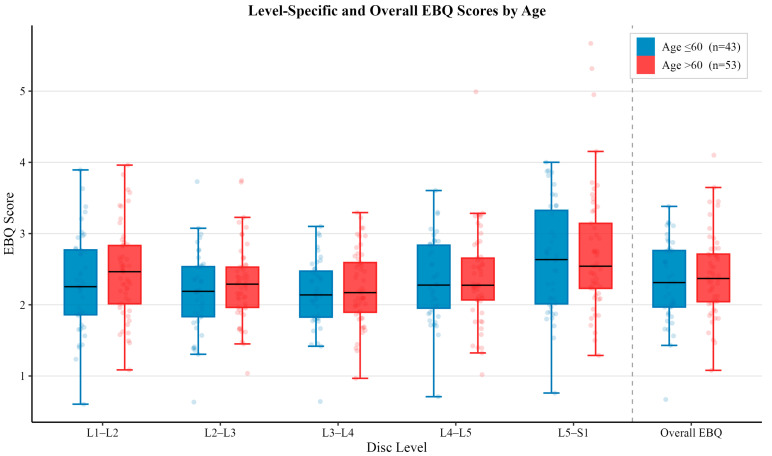
Comparison of mean EBQ scores between vertebral disc space level and overall EBQ between patients ≤ 60 years of age (*n* = 43) and > 60 years of age (*n* = 53). Error bars represent standard deviation. No statistically significant differences were observed between age groups for level-specific or overall EBQ score (*p* > 0.05).

For level-specific and comprehensive EBQ scores, there was no statistically significant difference observed between age associated subgroups (all *p* > 0.05). The average EBQ scores for patients ≤ 60 years of age for each lumbar level were 2.31 ± 0.69 (L1–L2), 2.17 ± 0.57 (L2–L3), 2.16 ± 0.48 (L3–L4), 2.34 ± 0.57 (L4–L5) and 2.63 ± 0.77 (L5–S1), with an average overall EBQ score of 2.32 ± 0.57. For patients > 60 years of age, the average level-specific EBQ scores were 2.46 ± 0.64 (L1–L2), 2.30 ± 0.53 (L2–L3), 2.24 ± 0.52 (L3–L4), 2.38 ± 0.66 (L4–L5), 2.74 ± 0.89 (L5–S1), and an average overall EBQ score of 2.42 ± 0.59. EBQ scores were higher for all level-specific and comprehensive scores for patients ≥ 60 years of age when compared to younger patients. The level-specific and comprehensive EBQ score differences for both subgroups are illustrated in Figure 2.

### 3.5. Sex Associated EBQ Scores

**Figure 3:**
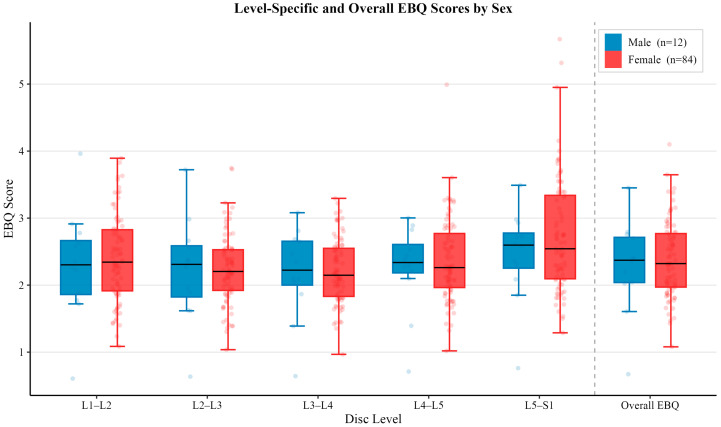
Comparison of mean EBQ scores between vertebral disc space level and overall EBQ between male (*n* = 12) and female (*n* = 84) patients. Error bars represent standard deviation. No statistically significant differences were observed between sex groups for level-specific or overall EBQ score (*p* > 0.05).

Similar to age stratification, no statistically significant difference was observed for level-specific and comprehensive EBQ scores between sex subgroups (*p* > 0.05). For males, the average EBQ score for each lumbar level was 2.31 ± 0.81 (L1–L2), 2.22 ± 0.78 (L2–L3), 2.18 ± 0.67 (L3–L4), 2.26 ± 0.65 (L4–L5) and 2.45 ± 0.69 (L5–S1), with an average overall EBQ score of 2.28 ± 0.69. The average level-specific EBQ scores for females were 2.40 ± 0.64 (L1–L2), 2.24 ± 0.52 (L2–L3), 2.21 ± 0.48 (L3–L4), 2.38 ± 0.62 (L4–L5), 2.73 ± 0.85 (L5–S1), and an average overall EBQ score of 2.39 ± 0.56. Additionally, the female subgroup demonstrated higher values for all level-specific and comprehensive EBQ scores compared to the male subgroup. The level-specific and comprehensive EBQ score differences for both subgroups are illustrated in Figure 3.

### 3.6. Bone Density Associated EBQ Scores

**Figure 4:**
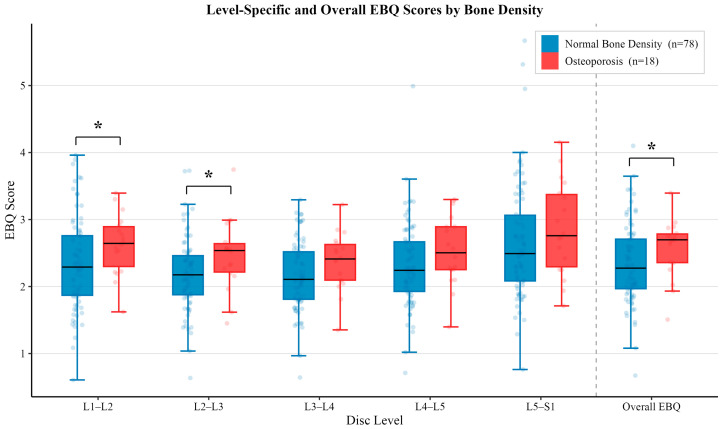
Comparison of mean EBQ scores between vertebral disc space level and overall EBQ between patients with normal bone density (*n* = 78) and osteoporosis (*n* = 18). Error bars represent standard deviation. Osteoporotic patients demonstrated significantly higher EBQ scores at the L1–L2 and L2–L3 levels and overall EBQ score (all *p* < 0.05), while no significant differences were observed at L3–L4 through L5–S1 (all *p* > 0.05). Asterisk (*) denotes significant difference between groups (*p* < 0.05).

There was a significant difference in overall EBQ score between osteoporotic patients (2.57 ± 0.44) and those with normal bone density (2.33 ± 0.60, *p* < 0.05). For level-specific analysis, EBQ scores for patients with normal bone density were 2.34 ± 0.69 and 2.19 ± 0.55, while scores for osteoporotic patients were 2.63 ± 0.45 and 2.47 ± 0.53 at the L1–L2 (*p* < 0.05) and L2–L3 (*p* < 0.05) levels, respectively. EBQ scores were 2.17 ± 0.51 at L3–L4, 2.32 ± 0.63 at L4–L5 and 2.65 ± 0.87 at L5–S1 for patients with normal bone density. These scores were not significantly different than those seen in osteoporotic patients, which were 2.37 ± 0.43 at L3–L4, 2.54 ± 0.52 at L4–L5 and 2.86 ± 0.69 at L5–S1. The level-specific and comprehensive EBQ score differences for both subgroups are illustrated in Figure 4.

## 4. Discussion

In patients with chronic lower back pain being considered for surgical intervention, MRI is the preferred modality for initial imaging [35]. As an MRI-derived metric, EBQ capitalizes on the T1-weighted slices of this existing imaging to assess the subchondral bone of the vertebral endplates and does not result in significant radiation exposure. By deriving bone quality metrics from imaging frequently obtained as part of the preoperative workflow, EBQ supports an opportunistic evaluation of the vertebral endplate. The integrity of the vertebral endplate is crucial for load bearing, with poor endplate bone quality implicated in cage subsidence following interbody fusion [36]. EBQ quantifies this region to provide information relevant to its integrity and the biomechanical challenges of interbody fusion. Despite the growing evidence supporting EBQ as a clinically meaningful bone quality assessment tool, all prior studies have measured EBQ exclusively at the operative level in surgical cohorts and for establishing procedure-specific thresholds. However, without a normative framework, interpreting these findings may be difficult. This study is the first to characterize level-specific EBQ scores throughout the entirety of the lumbar spine and provide normative reference data stratified by age, sex and osteoporosis, providing data to contextualize preoperative and patient-specific measurements.

The most clinically relevant finding of this study is the systematic caudal increase in EBQ scores, with the lowest values observed at L2–L3 and L3–L4, intermediate values at L1–L2, and the highest values at L5–S1. This gradient was consistent across both age groups and was observed in both sexes, suggesting it reflects an anatomic and biomechanical property of the lumbar spine rather than a characteristic of one demographic. The L4–L5 and L5–S1 segments are the most commonly targeted levels for lumbar interbody fusion, which may carry inherently higher EBQ values independent of pathology. Future studies evaluating the relationship between normative EBQ scores and thresholds derived from single-level surgical cohorts are needed to assess whether scores within the expected range at one level carry the same interpretation at another.

Osteoporotic patients demonstrated significantly higher EBQ scores at the L1–L2 and L2–L3 levels and in overall EBQ compared to patients with normal bone density. However, these differences were not observed at the L3–L4 through L5–S1 levels. This pattern may reflect the greater mechanical loading and degenerative endplate changes, leading to inherently increased EBQ values at caudal levels in healthy populations. Conversely, the lower EBQ scores at rostral levels may preserve sensitivity to osteoporotic changes. These findings suggest that patients diagnosed with osteoporosis may not demonstrate significantly different EBQ scores at more caudal levels than those seen in healthy individuals. These patients may be at increased risk for adverse postoperative events yet may remain undetected when utilizing EBQ alone. Further investigation of level-specific thresholds may be warranted to optimize EBQ interpretation across the lumbar spine.

Although statistically significant differences in level-specific or overall EBQ scores were observed between age subgroups or sexes, older patients and females demonstrated consistently higher EBQ values across all levels. The absence of statistical significance in these comparisons may reflect the sample size and the predominantly female cohort, which limit the detection of sex-specific differences. These trends are consistent with the established biology of bone quality decline with aging and the disproportionate burden of osteoporosis in women, warranting further investigation in more diverse cohorts.

Interrater reliability was good across all lumbar levels, with overall EBQ demonstrating an ICC of 0.76 and level-specific ICCs ranging from 0.70 at L3–L4 to 0.83 at L1–L2. The reduced reliability seen at L3–L4 may reflect the anatomic transition from lumbar vertebrae to more sacralized structures, where degenerative changes may influence ROI placement. This is consistent with the higher agreement at L1–L2, which frequently appear with classic features of lumbar vertebrae and may present with a more defined endplate morphology from lower degenerative burden. These reliability differences reinforce the importance of standardized segmentation protocols and highlight potential challenges in future clinical implementation.

By characterizing EBQ scores across all lumbar disc spaces rather than exclusively at the operative level, the results of this study may provide reference data for future studies evaluating patient-specific risk stratification. Prior studies have established that EBQ carries significant predictive value for cage subsidence across multiple surgical approaches, with reported AUC values ranging from 0.61 to 0.84 and optimal thresholds varying considerably by procedure [26,27,28,29,30,31,32,33,34]. This study may provide level-specific reference data for future studies comparing normative EBQ scores to patients whose EBQs exceed procedure-specific thresholds.

This study has several limitations. The single-institution, predominantly female cohort limits generalizability, though it does reflect the demographic most affected by osteoporosis. The retrospective design and absence of postoperative outcome data preclude direct correlation of level-specific EBQ patterns with adverse events such as cage subsidence or adjacent segment disease. Methodological heterogeneity across published EBQ studies, including variation in ROI shape, endplate thickness, and CSF sampling location, limits direct threshold comparisons and underscores the need for protocol standardization prior to clinical implementation. Additionally, the question of whether the current 3 mm endplate ROI captures the most biomechanically relevant portion of the endplate remains unresolved, as prior work has suggested that thinner ROIs may correlate more strongly with endplate volumetric BMD [27].

Future prospective, multi-institutional studies are needed to validate level-specific EBQ thresholds across surgical approaches and to correlate normative distributions with postoperative outcomes. The standardization of ROI dimensions, CSF sampling methodology, and endplate thickness should be established prior to broad clinical adoption.

## 5. Conclusions

EBQ represents a radiation-free technique to evaluate endplate bone quality with predictive value for cage subsidence in lumbar spine surgery; however, normative reference data remains limited. This study is the first to characterize level-specific EBQ distributions across the full lumbar spine. EBQ scores demonstrated a progressive caudal increase, with the lowest values at L2–L3 and L3–L4 and the highest at L5–S1, a pattern that was consistent across age and sex. Osteoporotic patients demonstrated significantly elevated EBQ at rostral levels, while caudal levels showed no significant difference, suggesting that inherently higher mechanical loading at L3–L4 through L5–S1 may reduce EBQ discriminative capacity at those levels in osteoporotic patients. Interrater reliability was acceptable across all levels, with reduced agreement at L3–L4 that may reflect its transitional anatomy. This study is the first to describe normative, level-specific EBQ scores across the lumbar spine. These findings may support future studies evaluating postoperative outcomes and threshold development.

## Figures and Tables

**Table 1:** Demographic and clinical characteristics of patients included in this study.

Characteristic	*n*
*Age, years (mean ± SD)*	61.0 ± 9.42
≤60	43 (44.8%)
>60	53 (55.2%)
*Sex*	
Female	84 (87.5%)
Male	12 (12.5%)
*Osteoporosis*	18 (18.8%)
*Smoking history*	36 (37.5%)
*Hypertension*	68 (70.8%)
*Diabetes mellitus*	29 (30.2%)
*Dyslipidemia*	30 (31.3%)
*Obesity*	41 (43.2%)
*VDD*	18 (18.8%)
*CKD*	12 (12.9%)
*Depression*	16 (16.7%)
*Anxiety*	27 (28.1%)

SD, standard deviation; VDD, Vitamin D deficiency; CKD, Chronic Kidney Disease.

**Table 2:** Summary of EBQ scores across levels overall and within subgroups.

Subgroup	*n*	*L1–L2*	*L2–L3*	*L3–L4*	*L4–L5*	*L5–S1*	*Overall*
Overall	96	2.39 ± 0.66	2.24 ± 0.55	2.21 ± 0.50	2.36 ± 0.62	2.69 ± 0.84	2.38 ± 0.58
≤60 Years	43	2.31 ± 0.69	2.17 ± 0.57	2.16 ± 0.48	2.34 ± 0.57	2.63 ± 0.77	2.32 ± 0.57
>60 Years	53	2.46 ± 0.64	2.30 ± 0.53	2.24 ± 0.52	2.37 ± 0.66	2.74 ± 0.89	2.42 ± 0.59
Males	12	2.31 ± 0.81	2.22 ± 0.78	2.18 ± 0.67	2.25 ± 0.65	2.45 ± 0.68	2.28 ± 0.69
Females	84	2.40 ± 0.64	2.24 ± 0.52	2.21 ± 0.48	2.37 ± 0.62	2.73 ± 0.85	2.39 ± 0.56
Osteoporosis	18	2.63 ± 0.45	2.47 ± 0.53	2.37 ± 0.43	2.54 ± 0.52	2.86 ± 0.69	2.57 ± 0.44
No osteoporosis	78	2.34 ± 0.69	2.19 ± 0.55	2.17 ± 0.51	2.32 ± 0.63	2.65 ± 0.87	2.33 ± 0.60

Values are presented as mean and standard deviation. EBQ, Endplate Bone Quality; BMD, bone mineral density; SD, standard deviation.

**Table 3:** Interrater reliability of Endplate Bone Quality measurement.

EBQ Score	MD (95% CI)	SDD	RMSE	r (*p*)	ICC (95% CI)
Overall	0.09 (0.004–0.18)	0.42	0.30	0.77 (<0.001)	0.76 (0.66–0.83)
L1–L2	0.09 (0.02–0.17)	0.39	0.28	0.84 (<0.001)	0.83 (0.76–0.89)
L2–L3	0.07 (−0.01–0.14)	0.38	0.27	0.79 (<0.001)	0.78 (0.69–0.85)
L3–L4	0.06 (−0.02–0.15)	0.42	0.30	0.71 (<0.001)	0.70 (0.59–0.79)
L4–L5	0.08 (−0.01–0.17)	0.46	0.44	0.75 (<0.001)	0.75 (0.64–0.84)
L5–S1	0.14 (0.02–0.26)	0.59	0.42	0.78 (<0.001)	0.77 (0.68–0.84)

EBQ, Endplate Bone Quality; MD, mean differences; SDD, standard deviation of differences; RMSE, root mean-squared error; r, Pearson correlation; ICC, interrater correlation coefficient.

## Data Availability

The data presented in this study are available on request from the corresponding author. The data presented in this study are restricted because it contains patient health information.

## Abbreviations

The following abbreviations are used in this manuscript:

DXA	Dual x-ray absorptiometry
BMD	Bone mineral density
EBQ	Endplate Bone Quality
MRI	Magnetic resonance imaging
VBQ	Vertebral Bone Quality
QCT	Quantitative computer tomography
SPA	Single-photon absorptiometry
DPA	Dual-photon absorptiometry
SXA	Single x-ray absorptiometry
SI	Signal intensity
MSC	Mesenchymal stem cell
CS	Cage subsidence
ROI	Region of interest
UEP	Upper endplate
LEP	Lower endplate
CSF	Cerebrospinal fluid
PLIF	Posterior lumbar interbody fusion
TLIF	Transforaminal lumbar interbody fusion
AUC	Area under curve
ASD	Adjacent segment disease
LLIF	Lateral lumbar interbody fusion
evBMD	Endplate volumetric bone mineral density
OLIF	Oblique lumbar interbody fusion
OLIF-PF	Oblique lumbar interbody fusion with posterior fixation
SA-OLIF	Standalone oblique lumbar interbody fusion
ICC	Interrater correlation coefficient
RMSE	Root mean squared error
r	Pearson correlation
CI	Confidence interval
T	Tesla
VDD	Vitamin D deficiency
CKD	Chronic kidney disease
SDD	Standard deviation of difference
MD	Mean difference
